# Low energy nebulization preserves integrity of SARS-CoV-2 mRNA vaccines for respiratory delivery

**DOI:** 10.1038/s41598-023-35872-4

**Published:** 2023-05-31

**Authors:** Cees J. M. van Rijn, Killian E. Vlaming, Reinout A. Bem, Rob J. Dekker, Albert Poortinga, Timo Breit, Selina van Leeuwen, Wim A. Ensink, Kelly van Wijnbergen, John L. van Hamme, Daniel Bonn, Teunis B. H. Geijtenbeek

**Affiliations:** 1 van der Waals-Zeeman Institute, Institute of Physics, University of Amsterdam, Amsterdam, The Netherlands; 2 Department of Experimental Immunology, Amsterdam University Medical Centers, Meibergdreef 9, Amsterdam, The Netherlands; 3 Institute for Infection and Immunity, Amsterdam University Medical Centers, Amsterdam, The Netherlands; 4 Pediatric Intensive Care Unit, Emma Children’s Hospital, Amsterdam University Medical Centers, Amsterdam, The Netherlands; 5 Swammerdam Institute for Life Sciences, University of Amsterdam, Amsterdam, The Netherlands

**Keywords:** Engineering, Biomedical engineering, Molecular medicine

## Abstract

Nebulization of mRNA therapeutics can be used to directly target the respiratory tract. A promising prospect is that mucosal administration of lipid nanoparticle (LNP)-based mRNA vaccines may lead to a more efficient protection against respiratory viruses. However, the nebulization process can rupture the LNP vehicles and degrade the mRNA molecules inside. Here we present a novel nebulization method able to preserve substantially the integrity of vaccines, as tested with two SARS-CoV-2 mRNA vaccines. We compare the new method with well-known nebulization methods used for medical respiratory applications. We find that a lower energy level in generating LNP droplets using the new nebulization method helps safeguard the integrity of the LNP and vaccine. By comparing nebulization techniques with different energy dissipation levels we find that LNPs and mRNAs can be kept largely intact if the energy dissipation remains below a threshold value, for LNP integrity 5–10 J/g and for mRNA integrity 10–20 J/g for both vaccines.

## Introduction

The COVID-19 pandemic has brought the field of RNA therapeutics to the foreground by the very large scale application of mRNA vaccines. Historically, the development of RNA formulations has been closely connected to that of lipid nanoparticles (LNPs), which are used to encapsulate the RNAs. The LNPs provide essential protection against extracellular RNases to avoid premature RNA degradation and enable efficient intracellular delivery. Of the various routes of administrating LNP-formulated RNA therapeutics^1,2^, inhalation promises important advantages^2,3^. It is expected to minimize systemic exposure and side effects, bypass renal or liver clearance, and avoid invasive injections. Furthermore, it could reduce the drug dose required to reach effective concentrations, as it is delivered directly to the large, well-perfused and highly immunological active surface area of the airways. Notably, mucosal targeting is thought to lead to a more efficient immunity against viruses such as SARS-CoV-2^4^. However, an important challenge for delivery of LNP-formulated mRNA by inhalation is due to the process used to produce small droplets that can be inhaled: nebulization can exert such shear stress on the LNPs that they rupture, thereby exposing the mRNA^5^ to omnipresent RNases in the extracellular space.

It is well documented that fluid-mechanical shear forces impact the integrity of very long nucleic acid molecules^6,7^. To achieve sustained vaccine-protein expression in cells, both the mRNA and LNPs should remain fully intact for as long as possible^8–10^. Thus, to enable inhaled RNA therapeutics, efforts have been made to strengthen the LNPs by using different lipid compositions for the LNP to survive the nebulization process^11^. Here we target on a reduction of LNP degradation by optimizing the nebulization method. We will not focus on respiratory mucus permeability of the LNPs, because the here used LNP mRNA vaccines have been solely optimized for intramuscular delivery.

**Figure 1:**
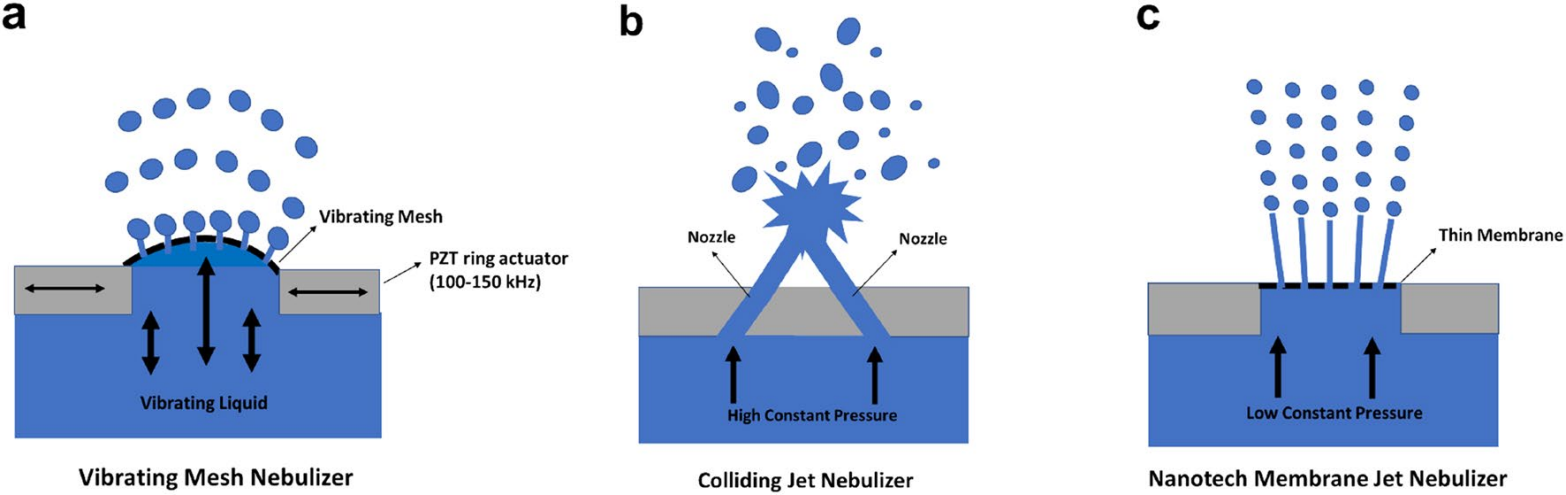
Methods for nebulization used in this study. (**a**) Vibrating mesh; a flexible mesh mounted on a piezoelectric lead zirconate titanate (PZT) ring actuator stretches and vibrates, thereby expelling droplets. (**b**) Colliding jet; two jets collide at a high velocity, herewith creating a Savart sheet that breaks up into small droplets. (**c**) Nanotech membrane; at low pressure, the fluid is pushed through a thin nanotech membrane with pores, herewith creating a plurality of equally sized jets that subsequently break up in equally sized droplets (Rayleigh breakup).

Figure 1 shows two existing nebulization techniques and a novel design that we will show has significant advantages over the other two in terms of safeguarding the integrity and biological activity of RNA therapeutics, in this case two SARS-CoV-2 mRNA vaccines: BNT162b (Comirnaty, BioNTech/Pfizer) and mRNA-1273 (mRNA-1273 Spikevax, Moderna). When considering nebulization techniques, it is important to realize that the droplet size range they produce critically determines where the therapeutics are deposited after inhalation. Droplets in the 0.5–4 µm range will be mainly deposited in the alveolar regions, droplets of 4–10 µm in the central and peripheral airways, and droplets larger than 10 µm mainly in the oral cavity, nose or throat^12^.

Of the techniques investigated, the vibrating mesh method (VM; Fig. 1a) is based on a flexible mesh mounted on a piezoelectric ring actuator (ultrasound transducer) that stretches and vibrates, thereby expelling droplets with sizes typically in the 1–10 µm range (Fig. S1). The vibrations however extend to the vaccine formulation and the LNPs they hold and are eventually dissipated by heat generation in the vaccine liquid, herewith increasing the temperature of the formulation in the reservoir during the nebulization process. The colliding jet (also called impinging jet) method (CJ; Fig. 1b) is based on collision at a large velocity of two jets, forming a thin liquid sheet at the location of impact (also known as a Savart sheet), that disintegrates into droplets, typically with a broad size range of 1–10 µm (Fig. S1). To ensure that the two jets have sufficient velocity to form droplets smaller than 10 µm after rupture of the Savart sheet, the required system pressure is typical between 150 and 200 bar. Finally, in the so-called nanotech membrane method (NM; Fig. 1c) the fluid is pushed through a thin nanofabricated membrane with about 50–100 equally sized nanoscale pores at a low pressure (5–20 bar), herewith creating a plurality of equally sized jets that subsequently break-up in droplets with a size peak in the range 2–5 µm (Fig. S1).

## Results and discussion

### Droplet size distribution of nebulized vaccine formulations

We investigated LNP composition before and after nebulization by determining the LNP diameter using dynamic light scattering (DLS). DLS primarily measures the Brownian motion of the LNPs in solution and relates this motion to the size of the LNPs. Samples were nebulized by two commercially available VM systems, a commercially available CJ system and the novel NM method (see Methods). The techniques differ in the energy input that is required to produce the droplets, as summarized in Table 1.

**Table 1:** Energy input required for nebulization.

Technique	Energy input (J/g)
Vibrating mesh, VM1	35 + /− 12
Vibrating mesh, VM2	18 + /− 6
Colliding jet, CJ	22 + /− 8
Nanotech membrane, NM	2 + /− 0.5

**Figure 2:**
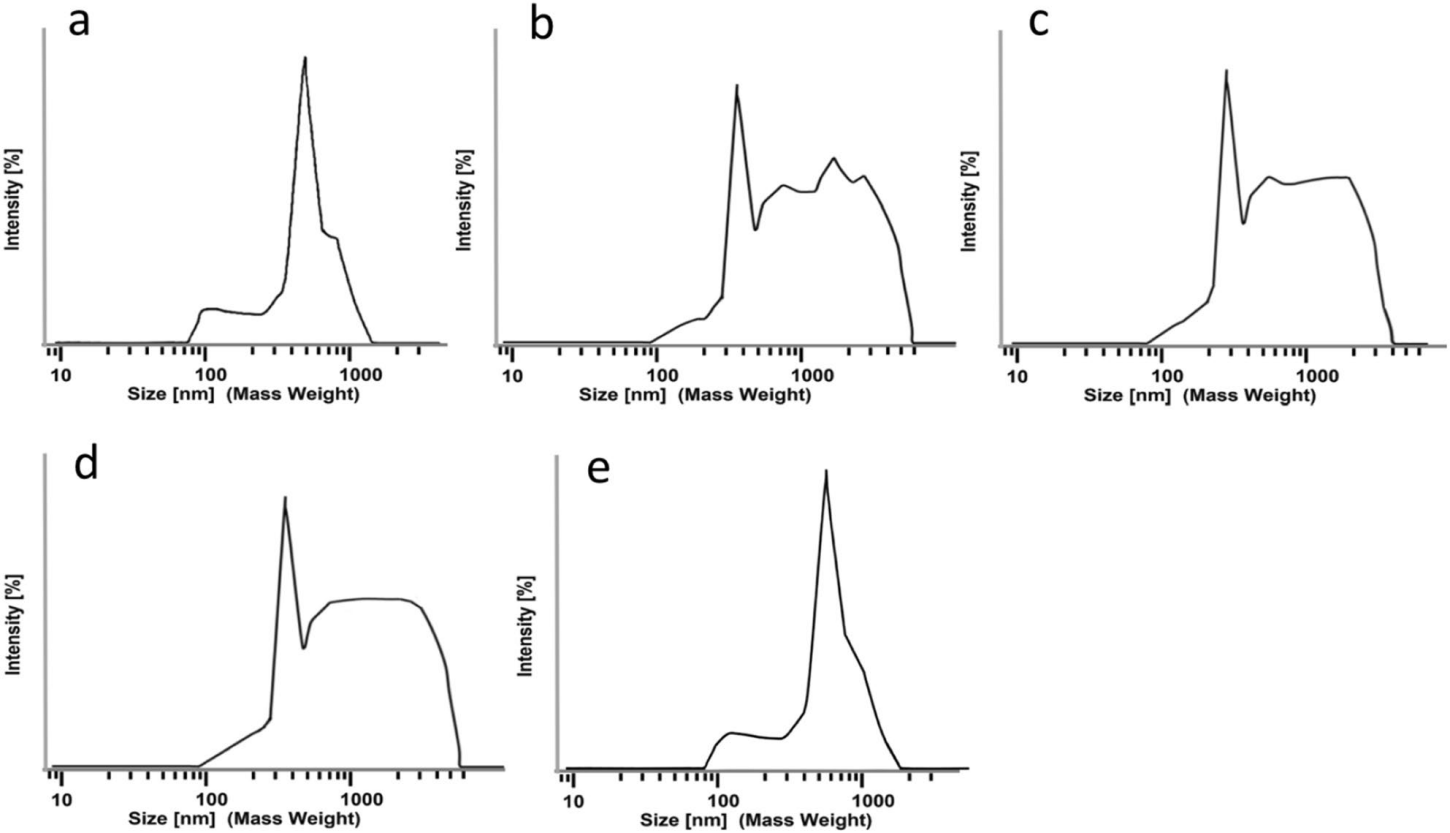
Particle size distributions of mRNA-1273 after nebulization, as obtained from Dynamic Light Scattering (DLS). (**a**) Stock formulation. (**b**) After nebulization with vibrating mesh VM1 at 35 J/g. (**c**) After nebulization with vibrating mesh VM2 at 18 J/g. (**d**) After nebulization with colliding jet (CJ) at 22 J/g. **e** After nebulization with nanotech membrane (NM) method at 2 J/g. Mass weight mode has been chosen to visualize the large particle fraction.

By eye, it was already clear that samples nebulized by the VM and CJ method were less transparent than the stock solution before nebulization. In contrast, the sample nebulized with the NM method retained its transparency. These observations indicate that nebulization by colliding jet and vibrating mesh techniques induce a change in LNP size distribution reflected by different light refraction, as confirmed by fully analyzing the DLS data (Fig. 2).

Figure 2a shows that the LNPs containing mRNA-1273 vaccine before nebulization have a size range of 300–500 nm. Nebulization by the VM and CJ methods causes an overall shift to larger LNP diameters (Fig. 2b–d), indicating aggregation of LNPs^13^, whereas nebulization by the NM method leaves the LNP size distribution largely unchanged (Fig. 2e). To investigate whether the larger LNPs are formed by aggregation of smaller or ruptured LNPs during nebulization, experiments were performed with a specific anti-aggregation formulation (Figs. S2,S3). Notably, a broad range of small to large LNPs were detected for the VM and CJ samples, indicating that the aggregates above 1 µm observed after nebulization can reasonably be assumed to be due to aggregation of smaller and ruptured LNPs.

### Impact of nebulization on mRNA-vaccine integrity

**Figure 3:**
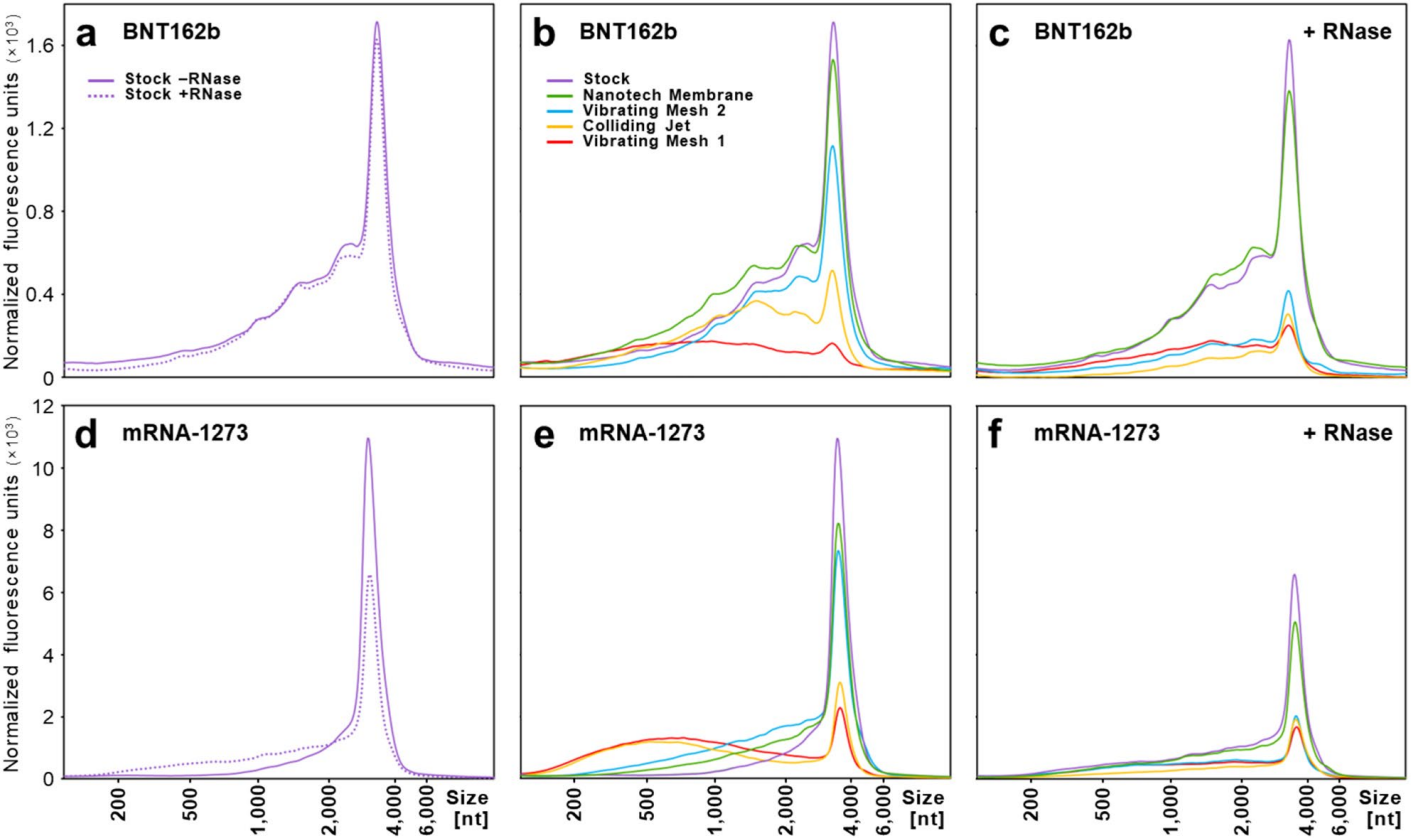
Effect of nebulization on mRNA integrity. Electropherograms are shown for the BNT162b (**a**–**c**) and mRNA-1273 (**d**–**f**) vaccines before and after nebulization with vibrating mesh type 1 (VM1; blue), colliding jet (CJ; yellow), vibrating mesh type 2 (VM2; red), and nanotech membrane (NM; green), all compared to the stock vaccines with and without RNase. Nebulized samples were treated with RNase to degrade all mRNAs not encapsulated by LNPs prior to electrophoretic analysis. The horizontal axis shows the RNA fragment size based on a series of RNA fragments with known lengths (200, 500, 1000, 2000, 4000 and 6000 nucleotides). For clarity, electropherograms were manually adjusted on the horizontal axis to align the full-length mRNA-vaccine peaks to the profiles obtained from the untreated stock solutions. The amount of RNA is shown as normalized fluorescence units on the vertical axis.

We next assessed the effect of the different nebulization methods on mRNA integrity of the two SARS-CoV-2 vaccines mRNA-1273 and BNT162b. The mRNA size of untreated and nebulized mRNA vaccines was analyzed by automated gel electrophoresis. The electropherograms of mRNA isolated from the non-nebulized samples (control condition) reveal a dominant peak between 3500 and 4000 for both vaccines (Fig. 3a,c), which is in line with the reported lengths of these mRNAs^14,15^.

Naked mRNA molecules are susceptible to both enzymatic (e.g. RNase) and non-enzymatic degradation (e.g. shear). To uncouple these two degradations mechanisms we studied RNA fragment lengths with and without adding RNase before after the nebulization process. RNase treatment before nebulization did not affect full-length mRNA of BNT162b (Fig. 3a) but decreased full-length mRNA from mRNA-1273 vaccine (dotted lines Fig. 3d). Notably, nebulization by the vibrating mesh 1 (VM1) and colliding jet (CJ) methods caused a 60–90% degradation of the mRNA in the vaccine (*P* < 0,001), as observed by the decrease of the full-length mRNA peak (Fig. 3b.e, Fig. S4, Table S1). In contrast, only a minor decrease (10–25%, *P* < 0,001) of full-length mRNA was observed after nebulization by the vibrating mesh 2 (VM2) and nanotech membrane (NM) methods. RNase treatment strongly decreased the full-length mRNA content of all nebulized samples, except the NM-nebulized samples (Fig. 3c,f). Strikingly, even though full-length mRNA seems not substantially affected by the VM2 method, the subsequent addition of RNase degraded the mRNA to a large extent, indicating that the LNPs were ruptured or dysfunctional after nebulization by the VM2 method. The resulting formulation was degraded to an extent comparable to the results of the VM1 and CJ method after adding RNase (Fig. 3c,f, Fig.S4). In contrast, after RNase treatment the nebulized samples obtained with the NM method showed much less mRNA reduction and remained comparable with the stock solutions, showing that there is hardly any degradation (BNT162b: 10.1 ± 1.4 and 7.7 ± 1.6 ng/μl; mRNA-1273: 33.8 ± 2.6 and 26.4 ± 2.7 ng/μl for stock and after NM-nebulization).

**Figure 4:**
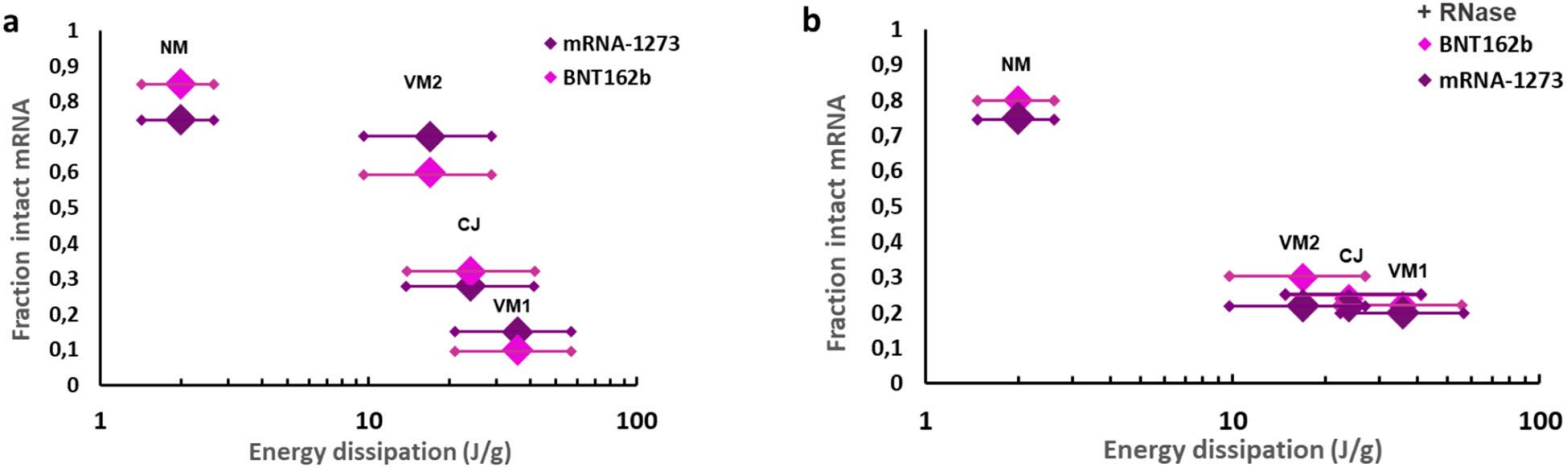
Fraction of intact mRNA versus nebulization energy dissipation. (**a**) without adding RNase. (**b**) with RNase added after nebulization.

To determine what energy levels involved in the nebulization method cause reversible deformation of the LNPs versus irreversible rupture, we plotted the fraction of mRNA remaining intact after nebulization as a function of the energy dissipation for all three nebulization methods of the two vaccine formulations with and without adding RNase (Fig. 4).

In Fig. 4a the fraction of intact mRNA is depicted without adding RNase after nebulization. The absence of RNase means that LNP rupture and subsequent breakage of the mRNA chains has solely occurred due to fluid mechanical shear forces. At an energy dissipation of about 10–20 J/g a substantial transition from intact to non-intact mRNA can be seen. In Fig. 4b the fraction of intact mRNA is depicted with added RNase after nebulization. This implies that LNP degradation is due to both fluid mechanical forces and enzymatic RNase activity. Here, a transition from intact to non-intact can be seen at an energy dissipation of about 5–10 J/g. Combining these figures, a picture emerges that LNPs start to rupture at an energy dissipation of about 5–10 J/g, releasing mRNA chains, and that fluid-mechanical breakage of these (naked) mRNA chains happens at energy densities exceeding about 10–20 J/g.

### Biological activity of nebulized mRNA vaccine

Next we investigated the biological activity of nebulized mRNA vaccines by determining the induction of SARS-CoV-2 Spike protein expression on the cell-surface of a human cell-line HEK293T after exposure to BNT162b and mRNA-1273, as both vaccines induce expression of transmembrane-anchored Spike protein^16,17^. K293T cells were incubated with vaccine stock or nebulized samples thereof. The expression of Spike proteins was measured after two days of culture by flow cytometry using anti-Spike antibody (COVA1-18) as the primary antibody and goat anti-human Alexa-488 antibodies as the secondary antibody.

**Figure 5:**
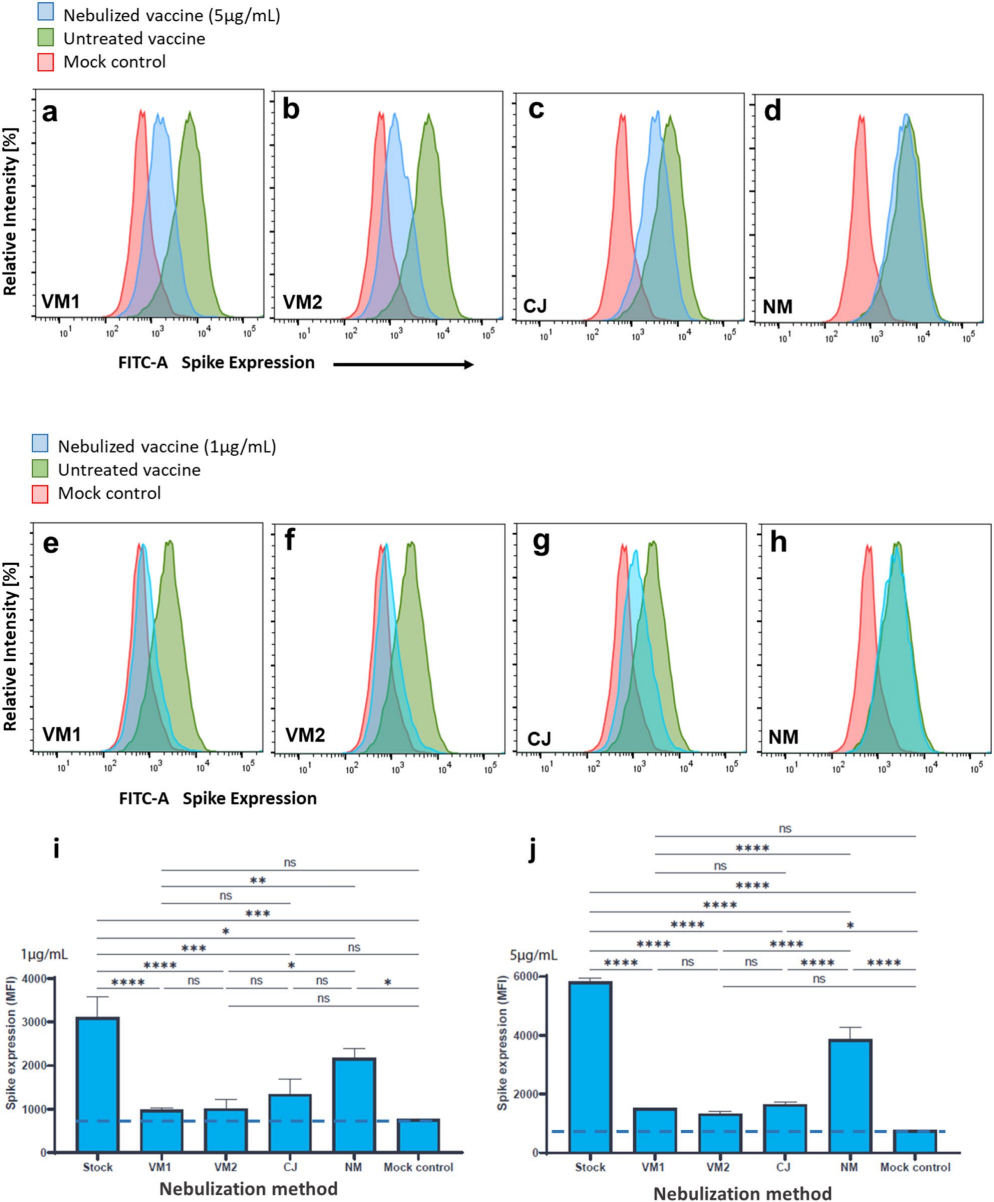
Impact of the various nebulization methods on the biological activity of BNT162b. (**a**–**h**) Experiments were performed in biological triplicates (stock solution, after nebulization, and Spike protein-free mock control) and plotted for statistical analysis at concentrations of 1 µg/ml and 5 µg/ml. (**i**, **j**) Statistical analysis using ordinary one-way ANOVA with Bonferroni multiple comparisons correction. ****p =  < 0,0001, ***p =  < 0,001, **p =  < 0,01, *p =  < 0,05, ns = not significant. Expression was quantified by mean fluorescent intensity (MFI). Blue dotted line represents the fluorescence background level as determined by Spike protein-free samples (mock control).

As shown in Fig. 5 for BNT162b, we find that nebulization of mRNA vaccine led to significantly lower expression of Spike proteins with samples nebulized using the vibrating mesh and colliding jet methods, whereas the nanotech membrane method outperforms the other two, with expression values far above the mock control level and close to pre-nebulization levels. Compared to the nanotech membrane method, the other nebulization methods induce around a two-fold larger decrease in Spike protein expression levels (Table S2). mRNA-1273 yielded very similar results (Fig. S5).

The decrease in Spike protein expression due to nebulization is quantitatively given in Table S2. Notably, treatment of the cells with the NM-nebulized samples resulted only in a minor decrease in Spike protein expression (1.43 and 1.51-fold for 1 µg/mL BNT162b (*P* = 0,03) and 5 µg/mL BNT162b (*P* < 0,0001), respectively; for mRNA-1273, the difference for 1 µg/mL was not significant whereas a 1.35-fold reduction was found for 5 µg/mL (*P* < 0,0001), see Table S2). In contrast, treatment of the cells with vaccine samples nebulized using the VM or CJ methods led to significant decreased (*i.e*., 2- to fourfold) induction of Spike protein expression as compared to untreated vaccines (Fig. 5*,* Table S2).

Mucosal immunity is paramount to prevent infection as well as spread by respiratory infections such as SARS-CoV-2^18^. Pulmonary and intranasal application of vaccines induces strong mucosal immunity^18,19^. Nebulization of vaccines is an efficient method for respiratory application. However, it remains unclear how nebulization affects the integrity of the vaccines in particular mRNA vaccines such as the COVID-19 mRNA vaccines. Our results reveal a consistent picture of how nebulization with increasing input energy levels first damage the LNPs carrying mRNA vaccine molecules and next the molecules themselves. Every form of degradation severely affects the biological activity of the vaccines administered in this way, counteracting the advantages offered by the inhalation technique. We confirmed that commonly used nebulization techniques induce significant degradation. To address this issue, we developed a low-energy nebulization technique based on a nanofabricated membrane, which causes much less degradation as a result of fluid-mechanical shear forces on the LNPs. By maintaining LNP integrity, the mRNA molecules are less susceptible post-nebulization to RNase, which is an important finding since RNases are omnipresent in the mucosal respiratory regions^18^ making LNP integrity vital for vaccine inhalation strategies.

Dissipation of energy has been shown before to be a determining factor in the break-up of dispersed structures such as emulsion droplets and liposomes under shear^20,21^. Other investigators have observed a threshold in shear above which liposomes and cells break^22,23^ and globular proteins unfold^24^. Kasaai et al*.* found a threshold energy density of 10 J/g for the break-up of chitosan molecules with a molecular weight of 2 MDa in water using a microfluidizer^25^. This molecular weight is close to the molecular weight of the mRNA used here (which has a length of 4,000 base pairs corresponding to a weight of about 1.3 MDa). Our measurements discern between rupture of the vaccine-carrying LNPs (at energy levels above 5–10 J/g) and fluid-mechanical breakage of mRNA chains (at energy densities above 10–20 J/g).

COVID-19 mRNA vaccines applied intramuscular are based on the transfection of cells with S protein, which leads to the induction of effective immune responses^19^. In order to study the mRNA vaccine integrity after nebulization, we applied the nebulized mRNA vaccines on HEK293T cells and investigated the induction of S protein. Notably, nebulization of the vaccines negatively affected the biological integrity. Especially nebulization by high vibrating mesh and colliding jet methods led to a strong decrease in S protein expression, whereas the low energy nanotech membrane method led to S protein expression levels close to those obtained with the non-nebulized vaccines. These data strongly suggest that low energy nebulization is a suitable method to retain the biological activity of mRNA vaccines.

Most inhalable vaccines administered via the nose are in the volume range of 0.01–0.05 ml^25^. The flow rate for the nanotech membrane method is about 2–3 ml/min. A volume of 0.05 ml with the nanotech method can then be administered by inhalation in about (0.05 ml/3.0 ml/60 s) 1–1.5 s by using e.g. a syringe with a nebulization chip (Fig. S6). Volumes of the current COVID19 mRNA vaccines for intramuscular administration however are between 0.25 and 0.3 ml with a dose of 30–100 ug mRNA. Such vaccine volumes would take about 5–8 s of inhalation time.

Here we have compared nebulization of mRNA vaccines by using a novel low energy nanotech membrane method and by using two classical high energy methods (colliding jet and vibrating mesh). High energy methods such as the vibrating mesh method have already been proven disruptive for certain LNPs^11,26^ and new more ‘robust’ LNP formulations have first to be developed when one wishes to use a high energy nebulization method. Our study shows that a low energy nebulization method seem more feasible. Even though the two tested mRNA vaccines have been approved for intramuscular injection and not mucosal delivery, our data strongly suggest that nebulization of COVID-19 vaccines via the low energy nanotech membrane method preserves very well both mRNA and LNP integrity. Therefore, the nanotech membrane nebulization method might also preserve the integrity of other mRNA vaccines optimized for mucosal delivery^26^ although this will need to be investigated further. Moreover, in vivo studies are needed to explore the full potential of inhaled LNP-mRNA formulations in the treatment and prevention of human diseases.

## Methods

### Nebulization methods

For the vibrating mesh (VM) technology, two commercially available nebulizers were used: VM1, a Pari eflow (Pari GmbH) and VM2, an Aerogen Solo (Aeroneb) nebulizer. The reservoirs of VM1 and VM2 were prefilled with 2–4 ml of the sample containing vaccine. The generated aerosols were collected in 30 mm diameter glass tubes and the collection time was measured for obtaining ca. 0.5–1 ml of nebulized fluid. A substantial increase in throughput with time (e.g., from 0.3 ml/min to 0.8 ml/min) was found for both vibrating mesh nebulizers, which is probably due to a temperature increase of the formulation due to dissipated vibration energy in the reservoir^21^. For the colliding jet (CJ) method, a Respimat (Boehringer Ingelheim) was used. A plastic cartridge of a reusable Respimat nebulizer was prefilled with the vaccine formulation and 60 doses or shots of 15µL each were collected in a glass tube. Each shot had a duration of about 2 s. Reloading the spring of the device also took about 2 s. For the nanotech membrane (NM) method nanotech membrane chips were made with silicon semiconductor technology. In a silicon body a free hanging silicon nitride membrane was made with a thickness of 800 nm and in this membrane 85 pores with a 1.9 nm diameter were made by reactive ion etching. A syringe (Fig S6) or spray pump was first prefilled with 1 ml vaccine formulation, which was manually pressed through the nanotech membrane nozzle at a pressure of 20 bar with a flowrate of 2–3 ml/min, and collected in a 10 mm diameter glass tube. All experiments were performed for both mRNA-1273 and BNT162b vaccines and reproduced three times. RNA degradation in the reservoirs before and after nebulization are given in Fig. S7.

### Biological activity of SARS-CoV-2 mRNA vaccines

We assessed the biological activity of the mRNA vaccines by transfection of HEK293T cells with the mRNA vaccines 20.000 HEK293T cells were seeded 24 h before treatment in 96 wells culture plates. mRNA-1273 (INN-COVID-19 mRNA vaccine, Moderna) was filtered through a 1 µm filter prior to nebulization using different nebulizers. The untreated filtered vaccine was used as a positive control. Following nebulization, the vaccine mRNA was incubated with the HEK293T cells for 48 h and expression of SARS-CoV-2 Spike proteins was determined by flow cytometry. Cells were harvested and incubated with human anti-Spike IgG COVA1-18 antibodies at 4 degrees for 30 min. Subsequently, cells were washed and incubated with a Goat anti-Human Alexa-488 (Invitrogen, Breda). Flow Cytometry analysis was subsequently performed on a BD FACS Canto II (BD Biosciences) and data was analyzed using FlowJo v10.8.1 (Software by Treestar). Statistical analysis and creation of graphs was subsequently performed using Graphpad Prisom 9 (Graphpad Software Inc.).

### mRNA isolation

To isolate pure vaccine mRNA, 300 μl Trizol LS reagent was added to the untreated samples. Phenol and guanidine isothiocyanate used in this Trizol LS method lyse the LNPs and protect the released vaccine mRNA against degradation by endonucleases. Next, the sample/Trizol LS mixtures were processed according to the manufacturer’s instructions until ± 230 μl of vaccine mRNA-containing water phase was obtained. The vaccine mRNA was purified and concentrated from the water phase into 12 μl using the RNeasy MinElute Cleanup Kit (Qiagen) according to the procedure described in Appendix D of the product manual. This RNA-isolation procedure was also done for nebulized samples and nebulized + RNase treated samples.

### mRNA quantification

Automated gel electrophoresis of the purified vaccine mRNA from all samples was performed on an Agilent 2200 TapeStation system using Agilent RNA ScreenTapes (Agilent). Electrophoresis profiles were rendered and quantified by the software Agilent Tape Station System Software (Rev. 4.1.1). The concentration of the full-length vaccine-mRNA moiety peak was quantified by manually defining regions tightly around the full-length vaccine-mRNA peak, preventing the inclusion of degraded vaccine mRNA as much as possible. The RNA concentration within the defined regions was calculated by the software and used as a measure for the concentration of full-length vaccine mRNA in the samples.

### RNase treatment

The freshly nebulized vaccine samples and stock vaccine samples (control condition) were split into two equal volumes of 100 µl. One subsample was treated for 5 min at 37 °C with RNase Cocktail Enzyme Mix (Thermo Fisher Scientific), containing a combination of RNaseA and RNaseT1 at a final concentration of 5 and 200 U/ml, respectively. This RNase treatment will completely degrade any vaccine mRNA that is not protected by intact LNP. After the RNase treatment, 300 μl Trizol LS reagent (Thermo Fisher Scientific) was added to the mixture to inactivate the RNases.

### Nebulization energy input

For the colliding jet nebulizer the spring constant of the spring to load the device was measured and from this, the energy stored in the spring and subsequently put into the sprayed formulation (15 μl) was directly calculated by using an energy transfer efficiency of 40–60%. For the nanotech membrane nebulizer, the amount of energy added to the spray was calculated from the pressure exerted on the piston of the syringe and the volume change. For the vibrating mesh nebulizer, the formulation was sprayed for 60 s and the temperature increase of the reservoir fluid was measured. This was done for three different amounts of reservoir fluid (1 g, 3 g and 6 g). The temperature increase was multiplied by the specific heat of water and the amount of liquid in the reservoir to arrive at the amount of energy taken up by the reservoir fluid during nebulization. The energy taken up by the reservoir fluid was extrapolated to a reservoir fluid amount of 0 g to get an indication of the amount of energy added to the sprayed fluid. This amount was divided by the amount of liquid that was sprayed to arrive at the energy density in J/g added to the sprayed fluid.

## Supplementary Information

Supplementary Information.

## Data Availability

The main data supporting the results in this study are available within the manuscript and Supporting Information. Data on mRNA degradation after nebulization and/or expression of spike proteins in selected cell lines generated during this study are available for research purposes on reasonable request from the corresponding author.
