# Author Correction: Low energy nebulization preserves integrity of SARS-CoV-2 mRNA vaccines for respiratory delivery

**DOI:** 10.1038/s41598-023-37515-0

**Published:** 2023-06-27

**Authors:** Cees J. M. van Rijn, Killian E. Vlaming, Reinout A. Bem, Rob J. Dekker, Albert Poortinga, Timo Breit, Selina van Leeuwen, Wim A. Ensink, Kelly van Wijnbergen, John L. van Hamme, Daniel Bonn, Teunis B. H. Geijtenbeek

**Affiliations:** 1 van der Waals-Zeeman Institute, Institute of Physics, University of Amsterdam, Amsterdam, The Netherlands; 2 Department of Experimental Immunology, Amsterdam University Medical Centers, Meibergdreef 9, Amsterdam, The Netherlands; 3 Institute for Infection and Immunity, Amsterdam University Medical Centers, Amsterdam, The Netherlands; 4 Pediatric Intensive Care Unit, Emma Children’s Hospital, Amsterdam University Medical Centers, Amsterdam, The Netherlands; 5 Swammerdam Institute for Life Sciences, University of Amsterdam, Amsterdam, The Netherlands

Correction to: *Scientific Reports* https://doi.org/10.1038/s41598-023-35872-4, published online 31 May 2023

The Acknowledgments section in the original version of this Article was omitted. The Acknowledgments section now reads:

“We thank Kaili Xi, Frank Verhoeven and Bernhard Müllinger for helpful discussions. Nanotech membrane chips were kindly provided by Medspray, the Netherlands.”

The original Article has been corrected.

