# Supplementary material for: Low energy nebulization preserves integrity of SARS-CoV-2 mRNA vaccines for respiratory delivery
Source: Sci Rep. 2023 May 31;13:8851. doi: 10.1038/s41598-023-35872-4 (PMC10231294; doi:10.1038/s41598-023-35872-4)
Supplement: Supplementary file 1 — Supplementary Information. [file 41598_2023_35872_MOESM1_ESM.docx]

**Supporting Information**

**Low energy nebulization preserves integrity of SARS-CoV-2 mRNA vaccines for respiratory delivery**

Cees JM van Rijn^1^, Killian E Vlaming^2, 3^, Reinout A Bem^4^, Rob J Dekker^5^, Albert Poortinga^1^, Timo Breit^5^, Selina van Leeuwen^5^, Wim A Ensink^5^, Kelly van Wijnbergen^2, 3^, John L van Hamme^2, 3^, Daniel Bonn^1,*^, Teunis BH Geijtenbeek^2, 3^

^1^ van der Waals-Zeeman Institute, Institute of Physics, University of Amsterdam,

Amsterdam, The Netherlands

^2^ Amsterdam UMC location University of Amsterdam, Department of Experimental Immunology, Meibergdreef 9, Amsterdam, The Netherlands

^3^ Amsterdam institute for Infection and Immunity, Amsterdam, The Netherlands

^4^ Pediatric Intensive Care Unit, Emma Children's Hospital,

Amsterdam University Medical Centers, The Netherlands

^5^ Swammerdam Institute for Life Sciences, University of Amsterdam, Amsterdam,

The Netherlands.

**Supporting Figures**

**
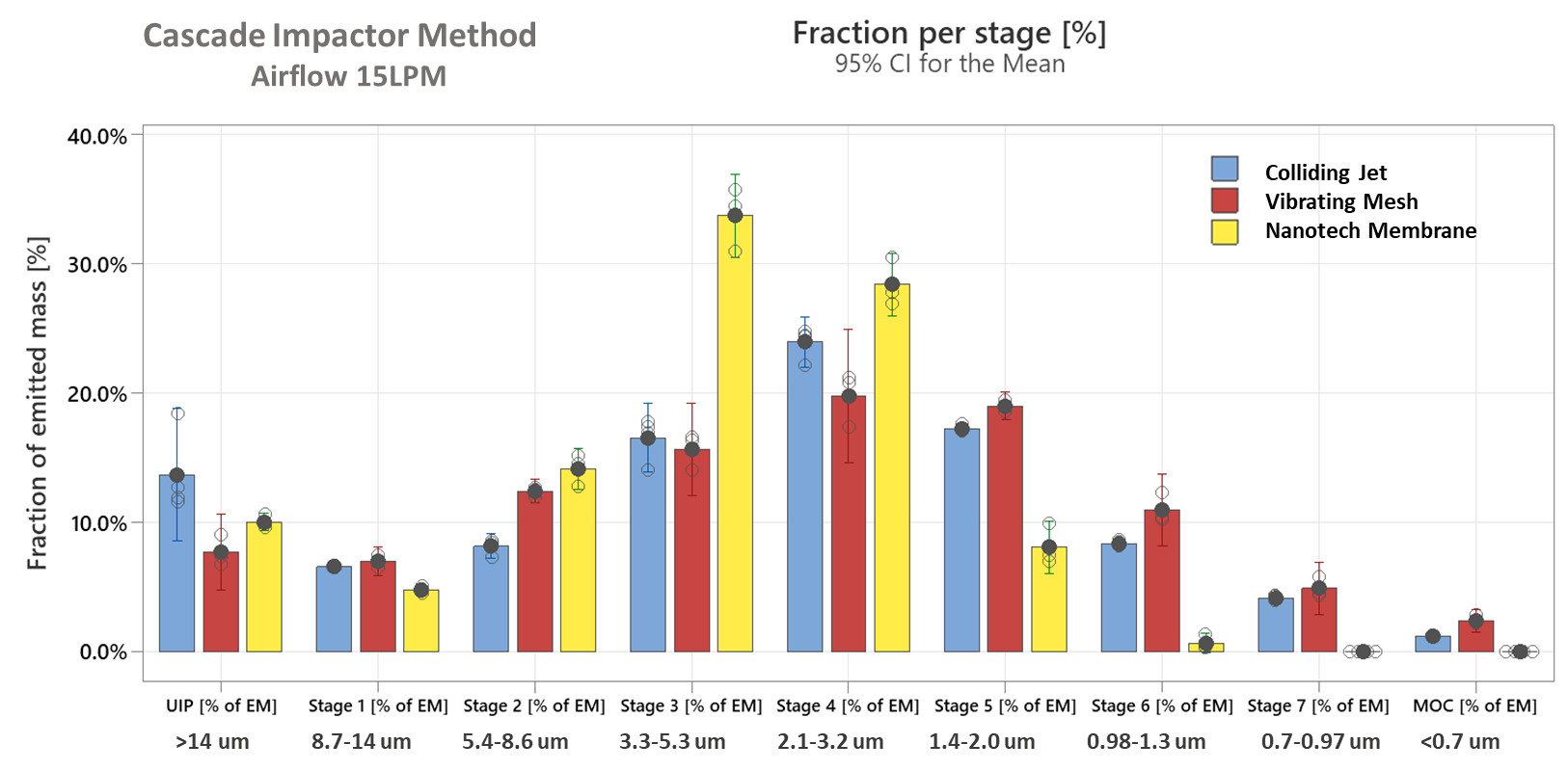
**

Fig. S1. Particle size distributions of the vibrating mesh, colliding jet and nanotech membrane nebulization method obtained with the 7 stage TSI Next Generation Impactor at an airflow of

15 LPM.


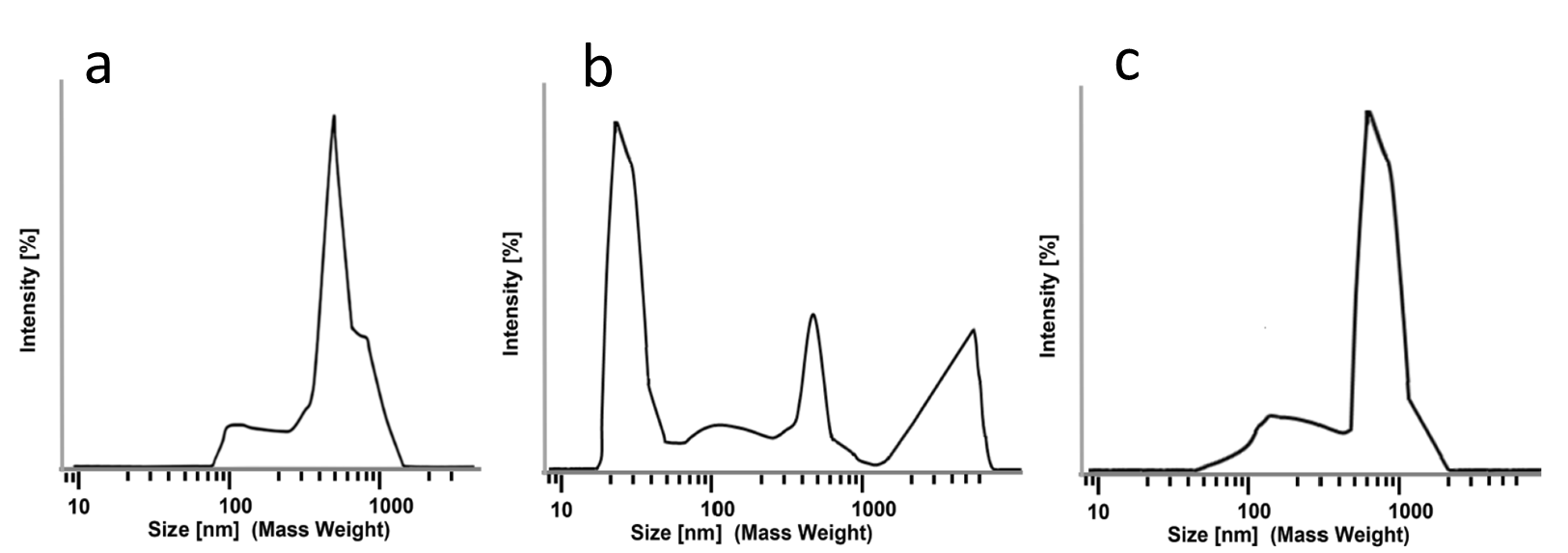


**Fig. S2. DLS spectra of mRNA-1273 with added tween20 (0.2%) before and after shear-induced fragmentation**. **a** Before nebulization. **b** After nebulization with vibrating mesh VM1. **c** After nebulization with nanotech membrane NM.


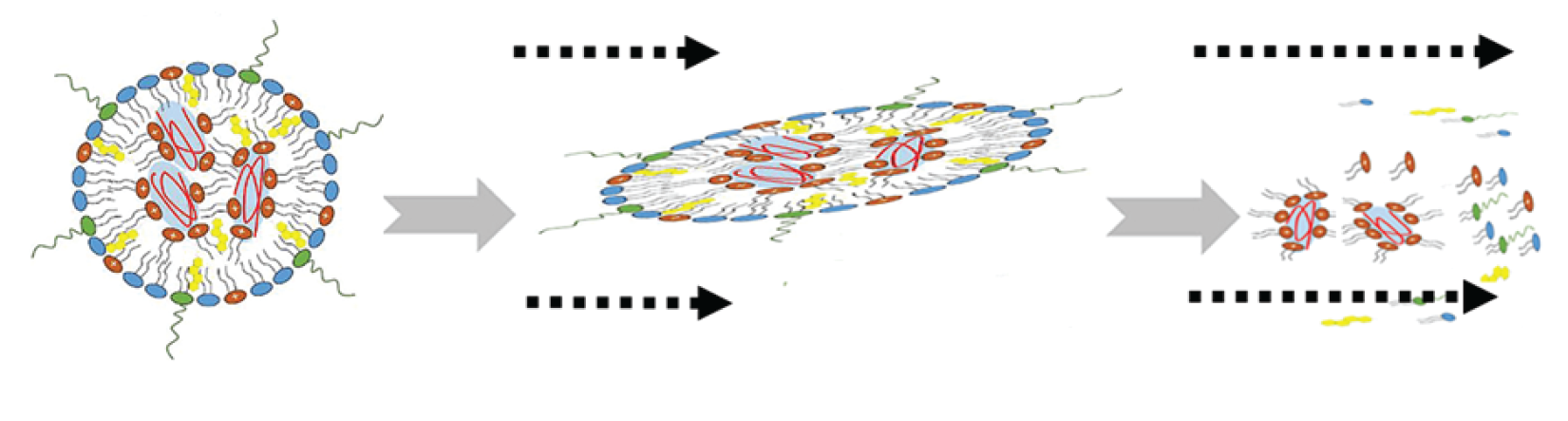


**Fig. S3. Schematic representation of shear-induced fragmentation of LNP-mRNA during nebulization.** Shown are mRNA chains and cationic lipids (red); neutral lipids (blue); PEGylated lipids (green), cholesterol (yellow), and fluid shear forces (dotted black arrows).


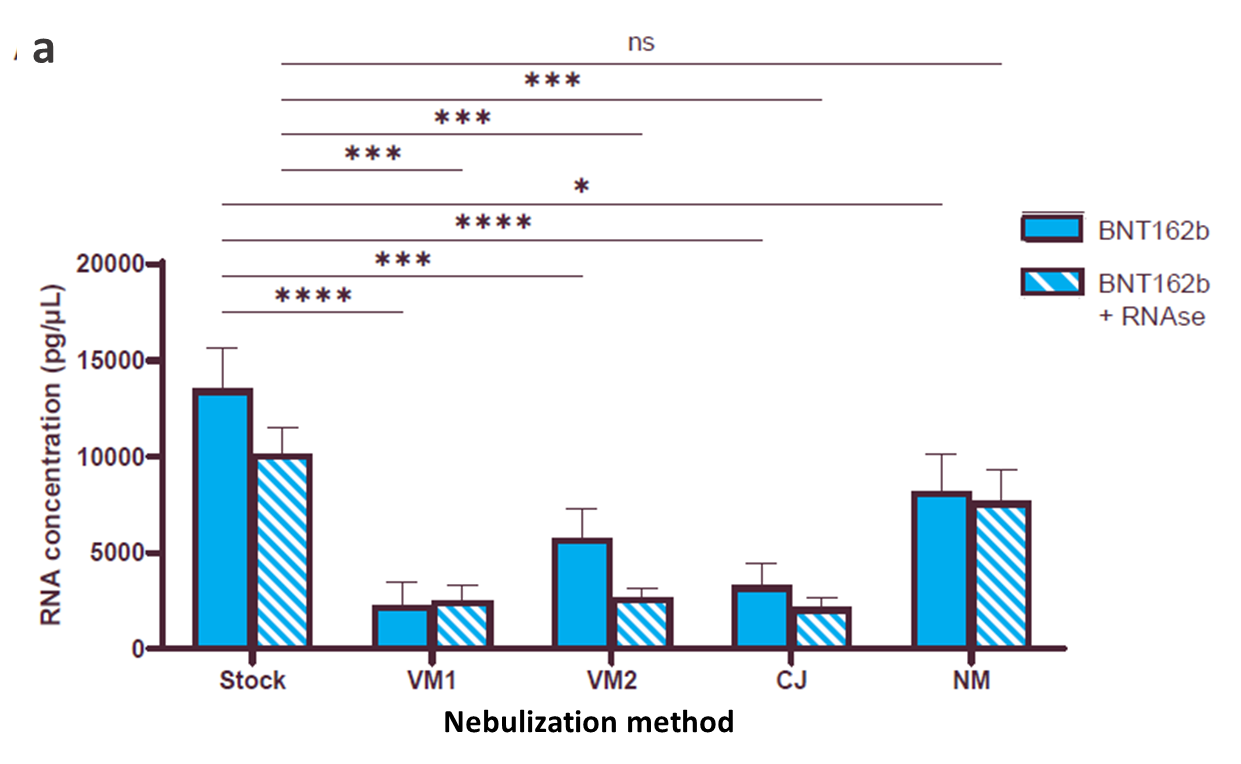

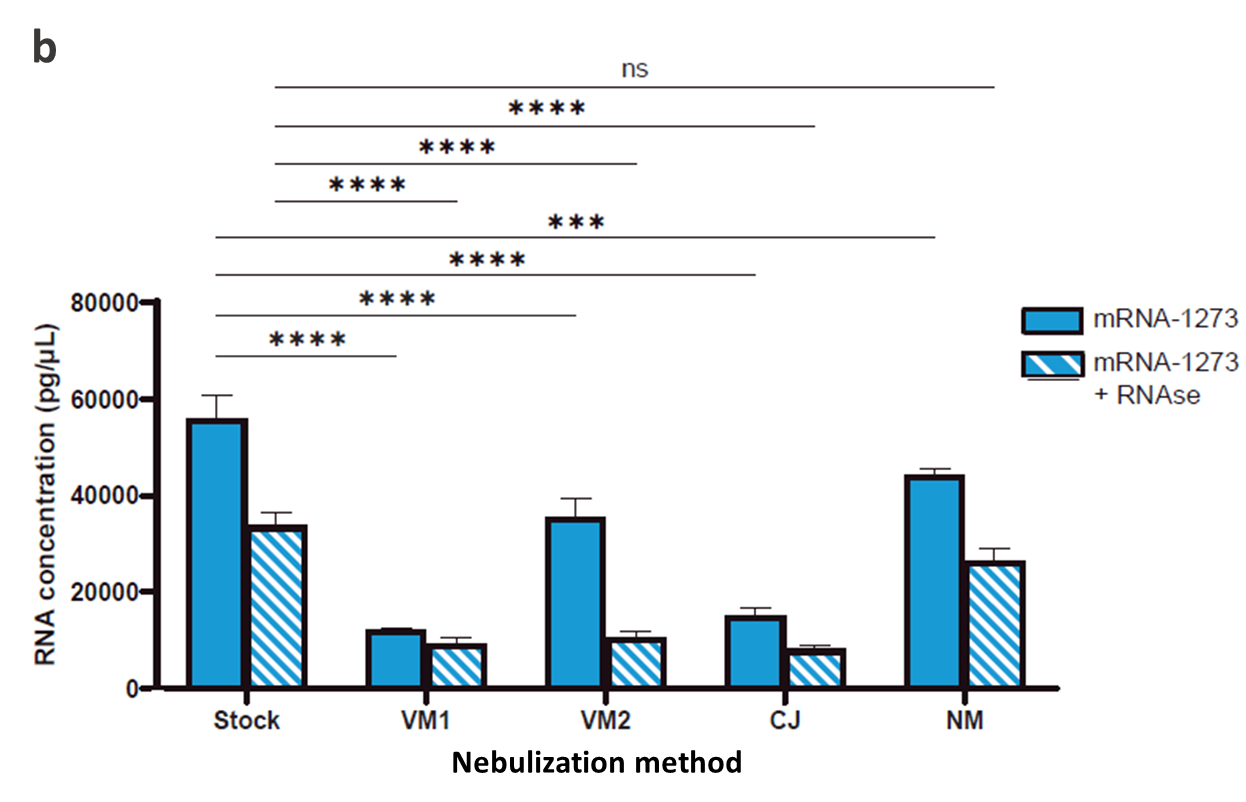


**Fig. S4. Impact of nebulization methods on LNP & mRNA integrity.** **a** concentration of the full-length vaccine mRNA for BNT162b and **b** for mRNA-1273. Results are shown after nebulization with vibrating mesh type 1 (VM1), vibrating mesh type 2 (VM2), colliding jet (CJ) and nanotech membrane (NM) methods, compared to the untreated stock vaccines. Gel electropherograms were used to quantify the concentration of full-length vaccine mRNA after nebulization by integrating the peak surface area. Concentrations were determined using the RNA molecular-weight size marker as a reference. The nebulized samples were either directly analyzed or after treatment with RNase to degrade all vaccine mRNA not encapsulated by LNPs. Average full-length vaccine mRNA concentrations (n = 3) in the diluted stock vaccines and nebulized vaccines are shown. Error bars indicate upper standard deviation. Statistical analysis was performed using a two-way ANOVA with Sidak’s multiple comparisons correction. ****p = <0,0001, ***p = <0,001, **p = <0,01, *p = < 0,05, ns = not significant, see also Table S1.


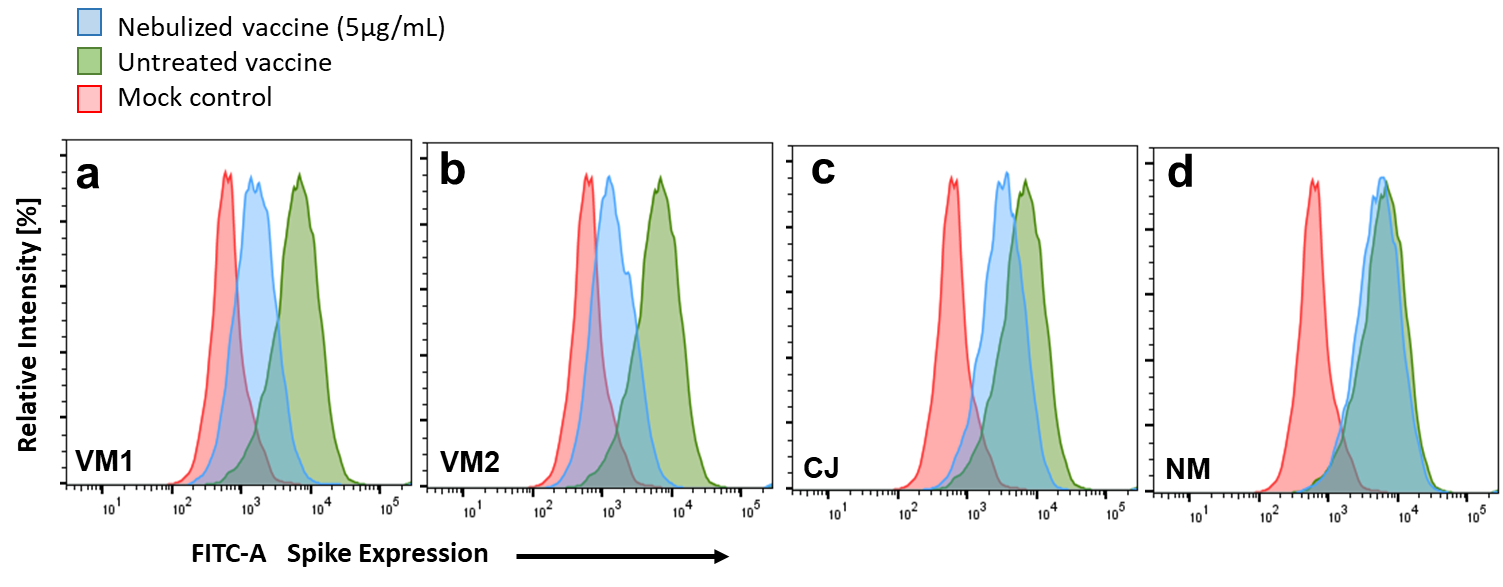


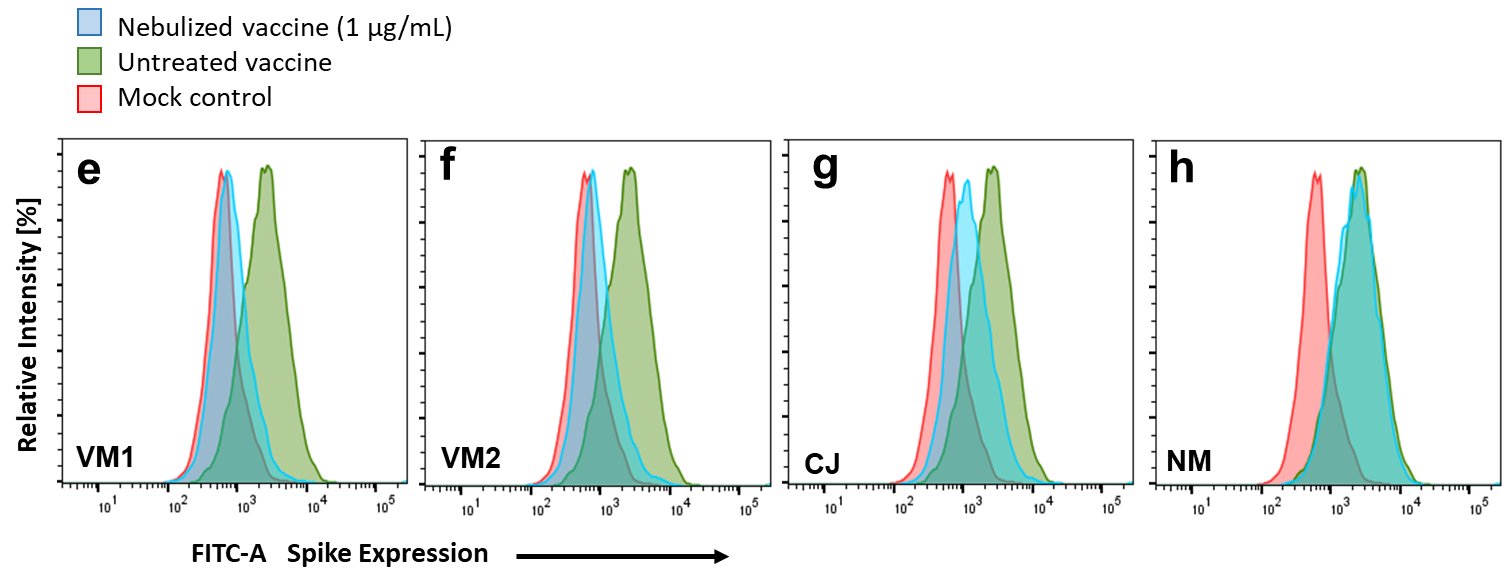


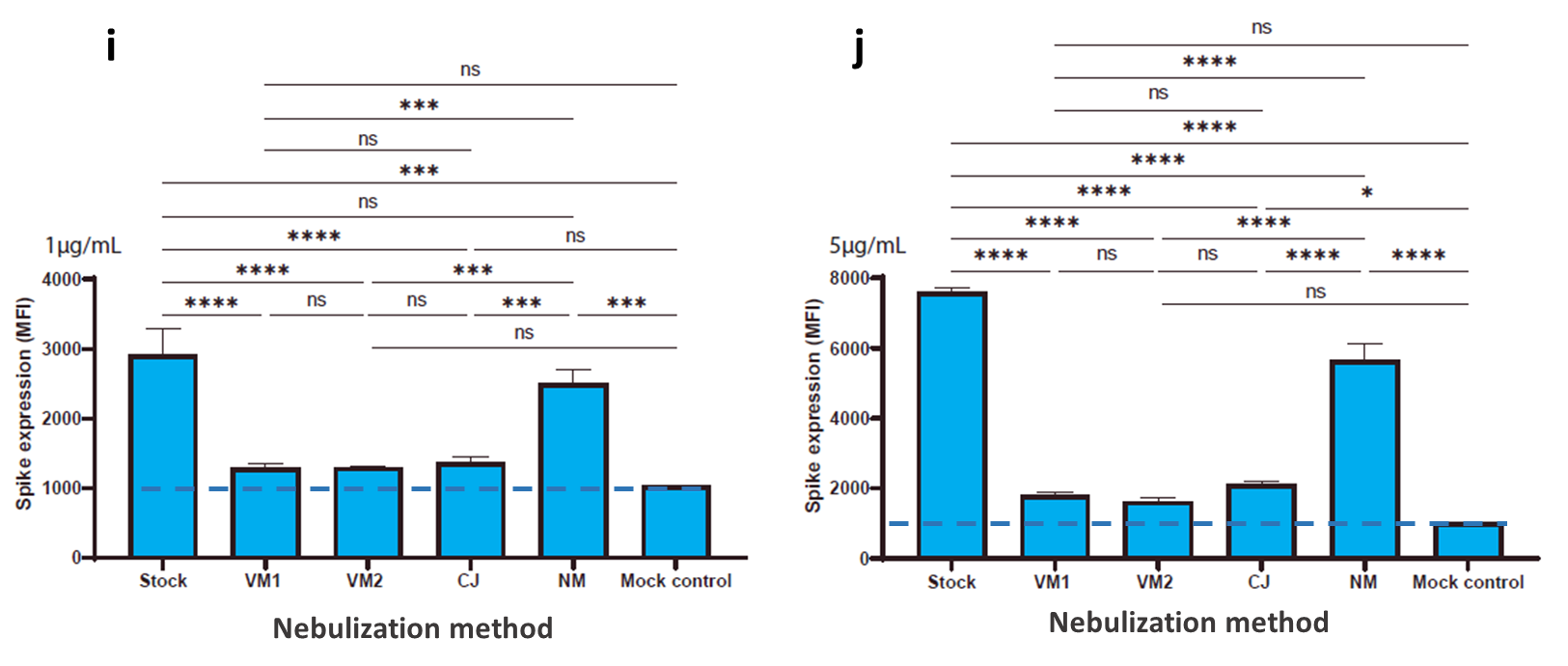


**Fig. S5. Impact of the nebulization method on the biological activity of mRNA-1273. a-d** concentration 5 µg/ml and **e-h** 1 µg/ml. Experiments were performed in biological triplicates (stock solution, after nebulization, and Spike protein-free mock control) and plotted for statistical analysis. **i, j** Statistical analysis was performed using ordinary one-way ANOVA with Bonferroni multiple comparisons correction. ****p = <0,0001, ***p = <0,001, **p = <0,01, *p = < 0,05, ns = not significant. Expression was quantified by mean fluorescent intensity (MFI). Blue dotted line represents the fluorescence background level as determined by Spike protein-free samples (mock control).


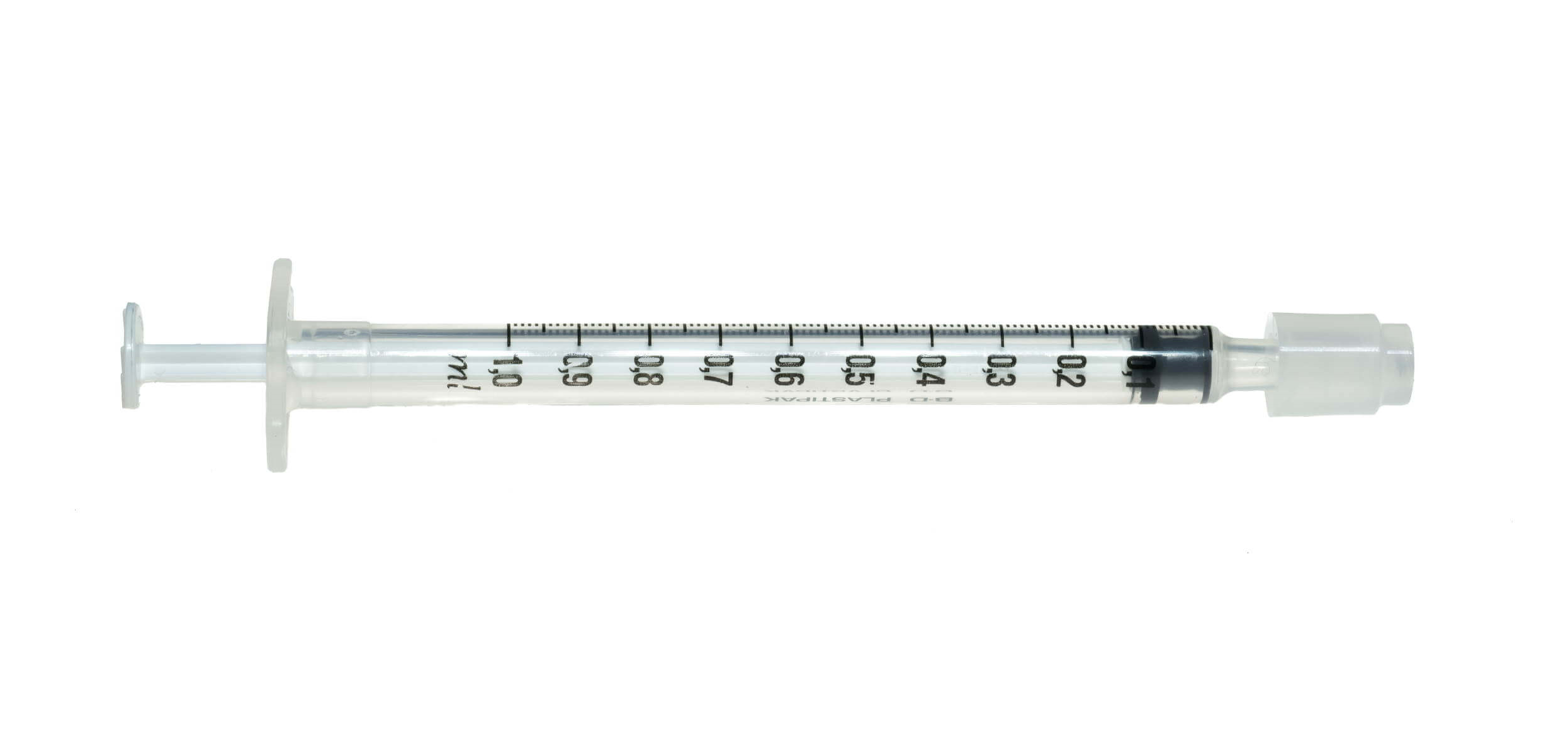

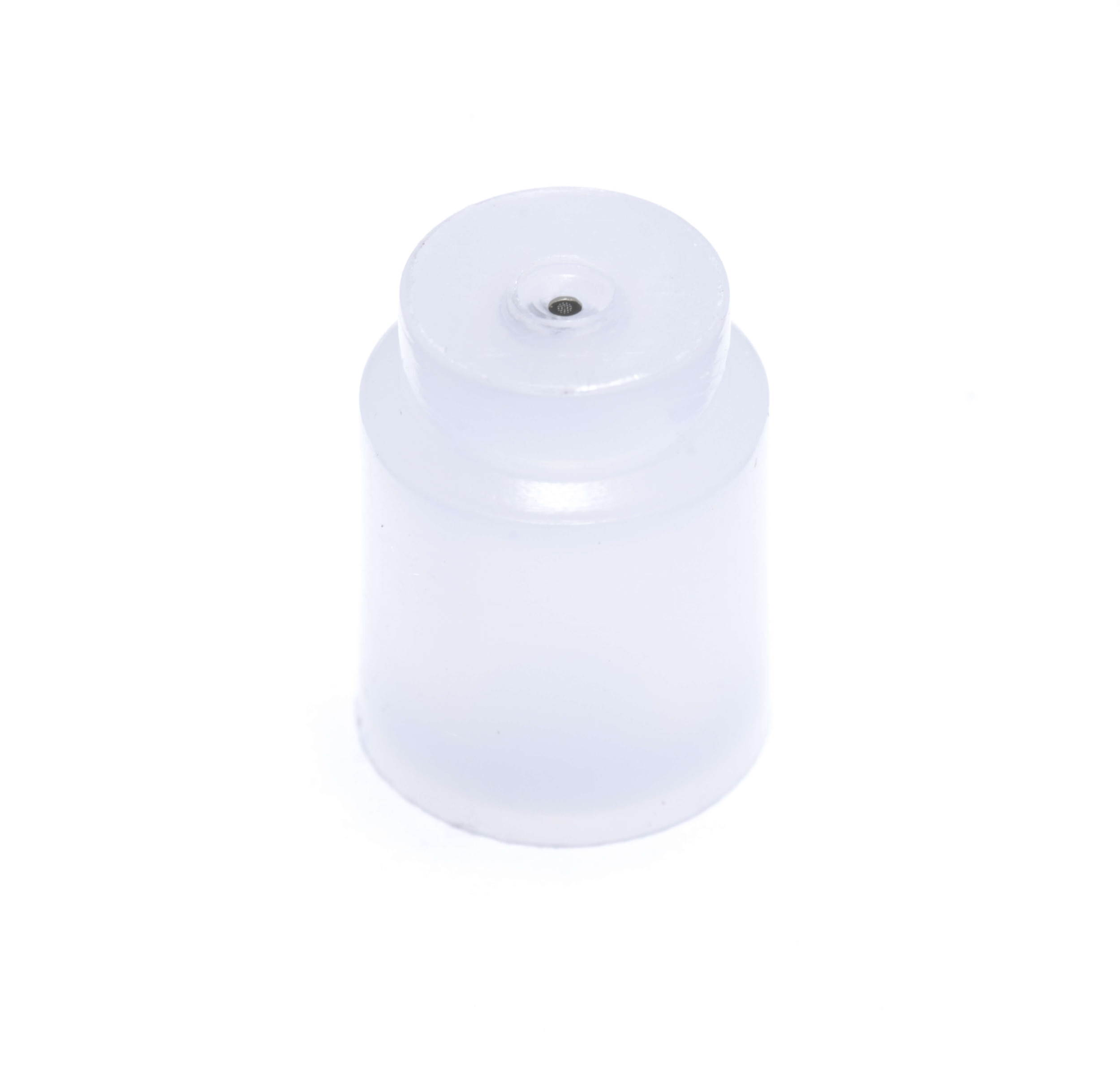


**Fig. S6. Nanotech membrane nebulization.** A syringe capped with a nanotech membrane nozzle as used in the experiments for evaluating the low energy nebulization method.


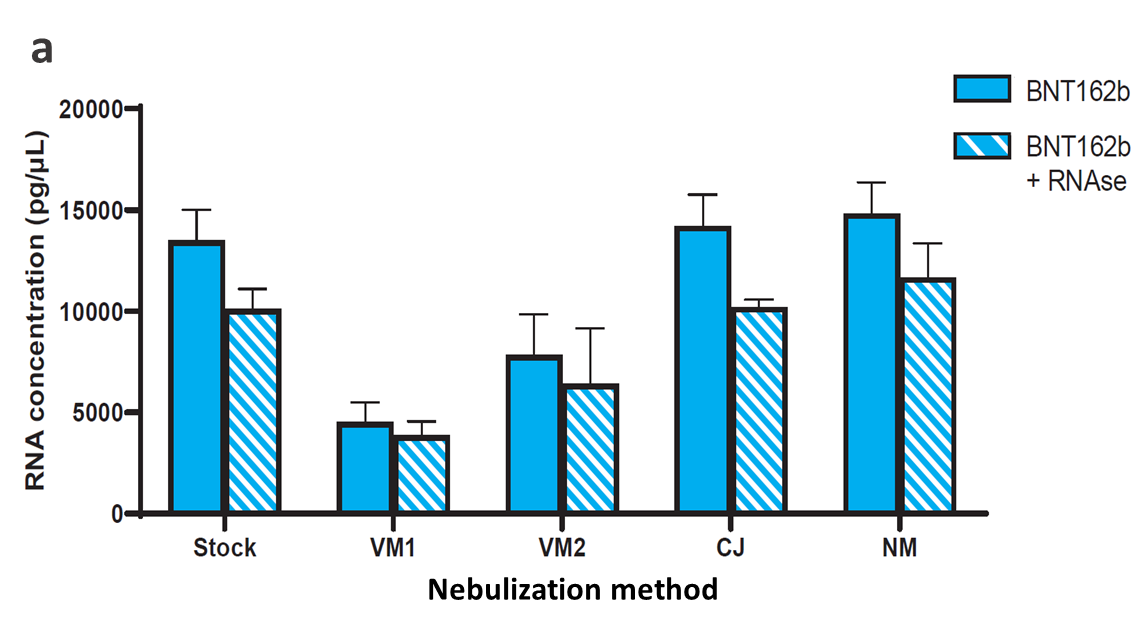

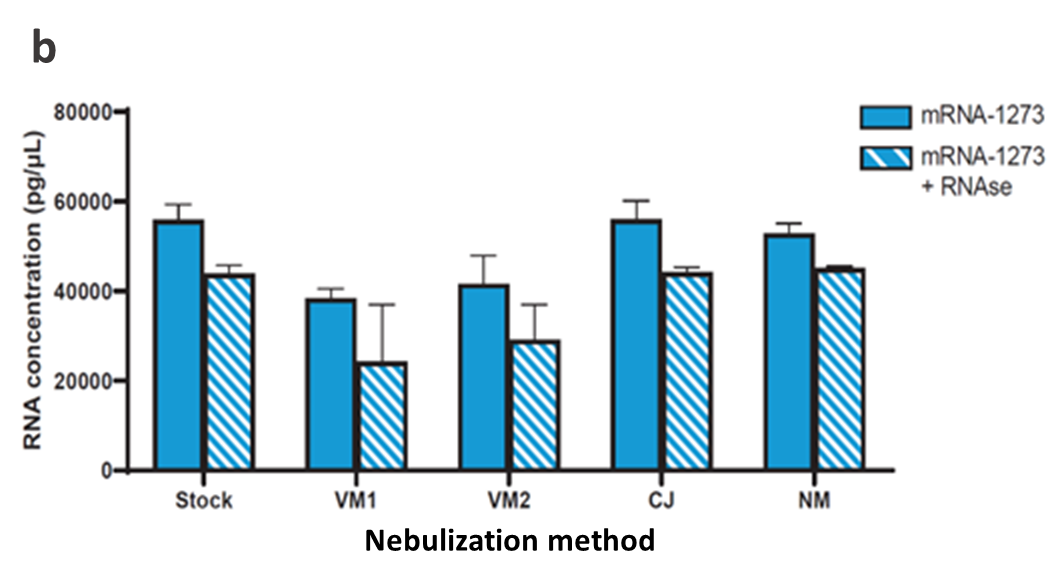


**Fig. S7. Impact of nebulization on the concentration of full-length vaccine mRNA in suspension within the reservoir**. Nebulization method is similar to what described in legend S4.

**a** concentration of the full-length vaccine mRNA for BNT162b and **b** for mRNA-1273. Results are shown after nebulization with vibrating mesh type 1 (VM1), vibrating mesh type 2 (VM2), colliding jet (CJ) and nanotech membrane (NM) methods, compared to the untreated stock vaccines. Gel electropherograms were used to quantify the concentration of full-length vaccine mRNA after nebulization by integrating the peak surface area. Concentrations were determined using the RNA molecular-weight size marker as a reference. The reservoir samples were either directly analyzed or after treatment with RNase to degrade all vaccine mRNA not encapsulated by LNPs. Average full-length vaccine mRNA concentrations (n = 3) in the diluted stock vaccines and nebulized vaccines are shown. Error bars indicate upper standard deviation.


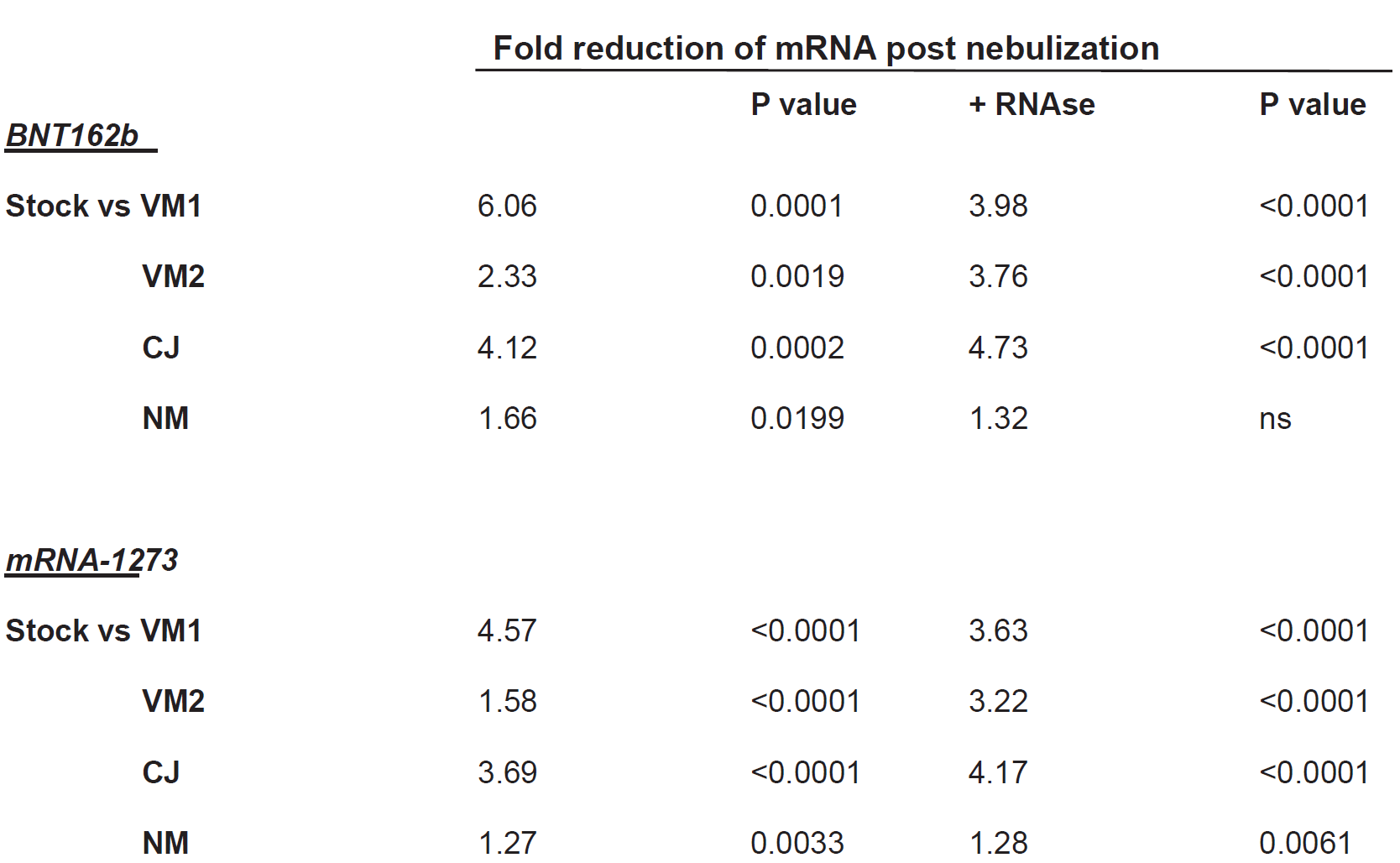


**Table S1.** Showing fold reduction observed in RNA concentrations following nebulization of BNT162b. Comparative reduction in RNA concentration following nebulization with various nebulizers and exposure to RNAse. Statistical analysis was performed using a two-way ANOVA with Sidak’s multiple comparisons correction. ****p = <0.0001, ***p = <0.001, **p = <0.01, *p = < 0.05, ns = not significant.


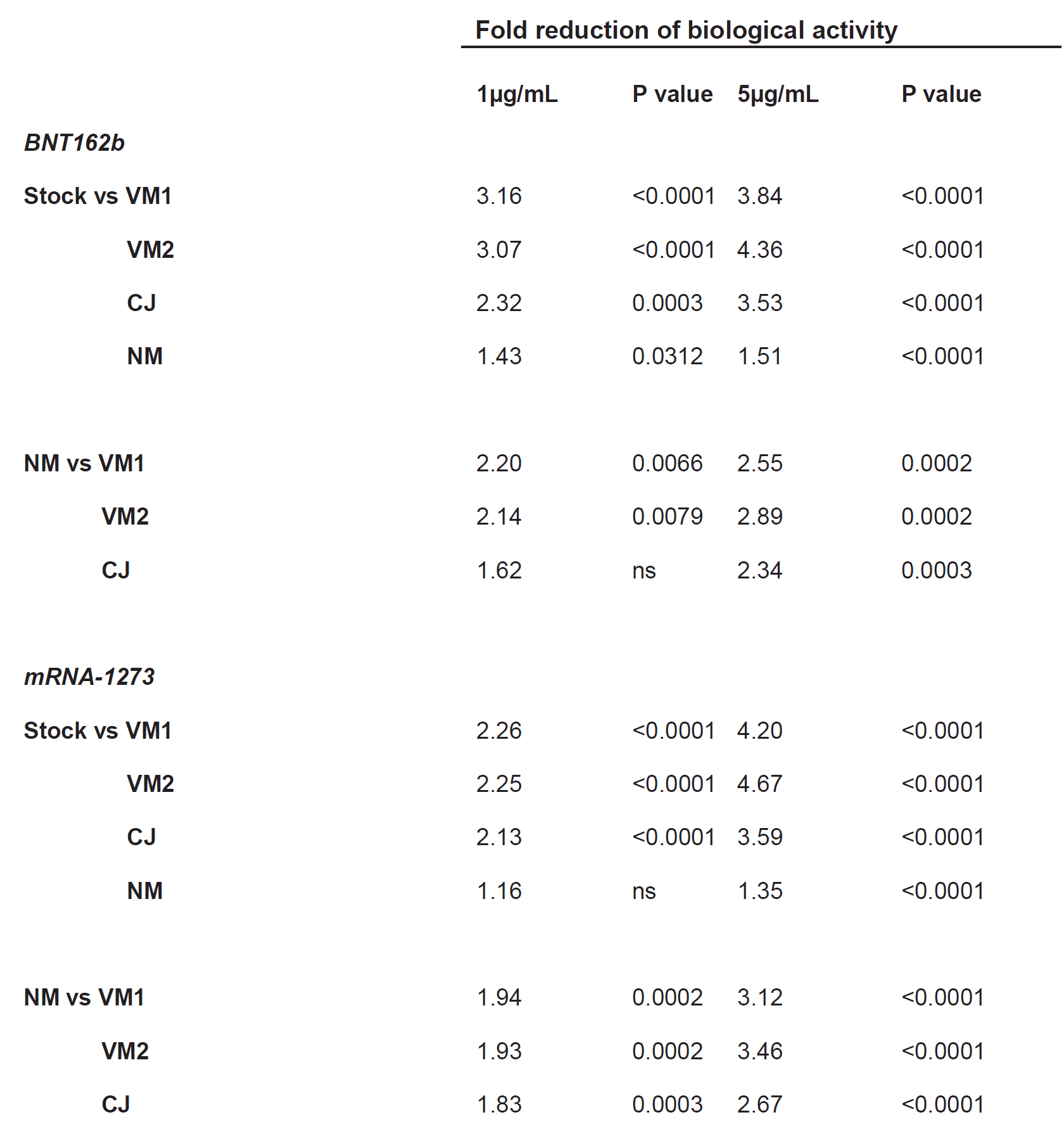


**Table S2.** Showing fold reduction in observed SARS-CoV-2 spike expression following nebulization for mRNA-1273 and BNT162b vaccines at different concentrations, using different nebulization methods.

**Materials**

**LNP mRNA vaccines**

Vials with 0.45 ml of the BNT162b (Comirnaty, BioNTech/Pfizer) vaccine and 1.5 ml of mRNA-1273 (mRNA-1273 Spikevax, Moderna) vaccine were obtained as a kind gift from the public health service of Amsterdam (GGD-Amsterdam) for research purposes. The BNT162b and mRNA-1273 vaccines were stored and prepared according to the instructions of the manufacturers and were additionally diluted just before usage with 25 ml of a sterile 0.9% NaCl solution, respectively. All samples were kept at 4°C at all times, except during nebulization.
